# Bis(μ-2-{1-[2-(dimethyl­amino)­ethyl­imino]­eth­yl}phenolato)bis­[bromido­copper(II)] monohydrate

**DOI:** 10.1107/S1600536811022045

**Published:** 2011-06-18

**Authors:** Nura Suleiman Gwaram, Hamid Khaledi, Hapipah Mohd Ali

**Affiliations:** a Department of Chemistry, University of Malaya, 50603 Kuala Lumpur, Malaysia

## Abstract

In the centrosymmetric dinuclear copper(II) title complex, [Cu_2_Br_2_(C_12_H_17_N_2_O)_2_]·H_2_O, each Cu^II^ ion is five coordinated in a square-pyramidal geometry by the *N,N′,O*-tridentate Schiff base, one Br atom and the bridging O atom of the centrosymmetrically related Schiff base. In the crystal, the water mol­ecules link the complex mol­ecules into infinite chains along the *b* axis *via* O—H⋯Br and C—H⋯O hydrogen bonds.

## Related literature

For the structures of some similar doubly bridged copper(II) complexes, see: Li *et al.* (2000 ▶); Rigamonti *et al.* (2008 ▶); Suo (2008 ▶). For a description of the geometry of complexes with five-coordinate metal atoms, see: Addison *et al.* (1984 ▶).
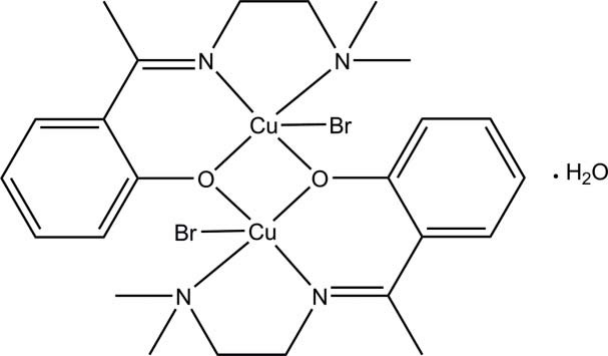


## Experimental

### 

#### Crystal data

[Cu_2_Br_2_(C_12_H_17_N_2_O)_2_]·H_2_O
*M* *_r_* = 715.47
Monoclinic, 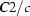

*a* = 20.754 (4) Å
*b* = 8.2492 (16) Å
*c* = 18.521 (4) Å
β = 119.528 (2)°
*V* = 2759.1 (9) Å^3^
*Z* = 4
Mo *K*α radiation
μ = 4.47 mm^−1^
*T* = 100 K
0.19 × 0.14 × 0.09 mm

#### Data collection

Bruker APEXII CCD diffractometer
Absorption correction: multi-scan (*SADABS*; Sheldrick, 1996 ▶) *T*_min_ = 0.484, *T*_max_ = 0.689
10414 measured reflections
3007 independent reflections
2623 reflections with *I* > 2σ(*I*)
*R*_int_ = 0.042

#### Refinement

*R*[*F* ^2^ > 2σ(*F* ^2^)] = 0.025
*wR*(*F* ^2^) = 0.060
*S* = 1.05
3007 reflections
165 parameters
1 restraint
H atoms treated by a mixture of independent and constrained refinement
Δρ_max_ = 0.36 e Å^−3^
Δρ_min_ = −0.51 e Å^−3^

### 

Data collection: *APEX2* (Bruker, 2007 ▶); cell refinement: *SAINT* (Bruker, 2007 ▶); data reduction: *SAINT*; program(s) used to solve structure: *SHELXS97* (Sheldrick, 2008 ▶); program(s) used to refine structure: *SHELXL97* (Sheldrick, 2008 ▶); molecular graphics: *X-SEED* (Barbour, 2001 ▶; Atwood & Barbour, 2003 ▶); software used to prepare material for publication: *SHELXL97* and *publCIF* (Westrip, 2010 ▶).

## Supplementary Material

Crystal structure: contains datablock(s) I, global. DOI: 10.1107/S1600536811022045/ez2243sup1.cif

Structure factors: contains datablock(s) I. DOI: 10.1107/S1600536811022045/ez2243Isup2.hkl

Additional supplementary materials:  crystallographic information; 3D view; checkCIF report

## Figures and Tables

**Table 1:** Hydrogen-bond geometry (Å, °)

*D*—H⋯*A*	*D*—H	H⋯*A*	*D*⋯*A*	*D*—H⋯*A*
C11—H11*B*⋯O2^i^	0.98	2.40	3.299 (3)	152
O2—H2*O*⋯Br1	0.83 (2)	2.62 (2)	3.4269 (14)	167 (3)

Symmetry code: (i) 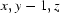.

**Fig. 1.**
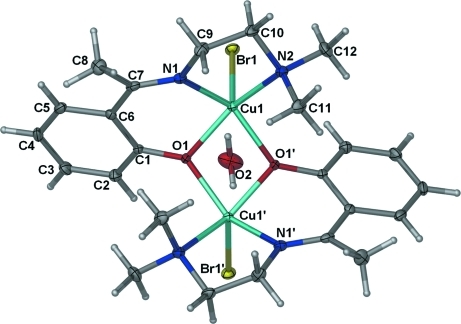
Thermal ellipsoid plot of the title compound at the 50% probability level. Hydrogen atoms are drawn as spheres of arbitrary radius. Symmetry code: ' = -x, y, -z + 1/2.

## supplementary crystallographic information

### Comment

The title dimeric copper(II) complex was synthesized through the reaction of the *in situ* prepared Schiff base, *N,N*-dimethyl-*N'*-[methyl(2-phenolyl)methylene]ethane-1,2-diamine, with copper(I) bromide. Under the reaction conditions, the Cu^I^ ion was oxidized to Cu^II^ and chelated by the deprotonated *N,N',O*-tridentate Schiff base. Pairs of metal centers are doubly bridged *via* the phenoxide O atoms around centers of inversion. Within the formed dimer, the Cu···.Cu distance [2.9935 (8) Å] is comparable to those reported for similar structures (Li *et al.*, 2000; Rigamonti *et al.*, 2008; Suo, 2008). The square-pyramidal geometry (*τ =* 0.11, Addison *et al.*, 1984) around each Cu^II^ ion is completed by one apically positioned Br atom. The dimeric complex is cocrystallized with one molecule of water whose oxygen atom is situated on a 2-fold rotational axis. In the crystal, the water molecules link the dimers into infinite chains along the *b* axis *via* O—H···Br and C—H···O interactions.

### Experimental

A solution of 2-acetylpyridine (0.20 g, 1.65 mmol) and *N*,*N*-dimethylethyldiamine (0.14 g, 1.65 mmol) in ethanol (20 ml) was stirred at reflux for 2 hr. Then, a solution of copper (I) bromide (0.21 g, 1.65 mmol) in a minimum amount of ethanol was added. The resulting mixture was refluxed for 30 min, and then left at room temperature. The crystals of the title complex were obtained in a few days.

### Refinement

The C-bound H atoms were placed at calculated positions at distances C—H = 0.95, 0.98 and 0.99 Å for aryl, methyl and methylene type H-atoms, respectively. The O-bound H atom was placed in a difference Fourier map, and was refined with distance restraint of O—H 0.84 (2) Å. For all hydrogen atoms U*iso*(H) were set to 1.2–1.5 times U*eq*(carrier atom).

### Crystal data

**Table:**

[Cu_2_Br_2_(C_12_H_17_N2O)_2_]·H_2_O	*F*(000) = 1440
*M**_r_* = 715.47	*D*_x_ = 1.722 Mg m^−^^3^
Monoclinic, *C*2/*c*	Mo *K*α radiation, λ = 0.71073 Å
Hall symbol: -C 2yc	Cell parameters from 3159 reflections
*a* = 20.754 (4) Å	θ = 2.4–30.5°
*b* = 8.2492 (16) Å	µ = 4.47 mm^−^^1^
*c* = 18.521 (4) Å	*T* = 100 K
β = 119.528 (2)°	Block, green
*V* = 2759.1 (9) Å^3^	0.19 × 0.14 × 0.09 mm
*Z* = 4	

### Data collection

**Table:**

Bruker APEXII CCD diffractometer	3007 independent reflections
Radiation source: fine-focus sealed tube	2623 reflections with *I* > 2σ(*I*)
graphite	*R*_int_ = 0.042
φ and ω scans	θ_max_ = 27.0°, θ_min_ = 2.3°
Absorption correction: multi-scan (*SADABS*; Sheldrick, 1996)	*h* = −26→26
*T*_min_ = 0.484, *T*_max_ = 0.689	*k* = −10→10
10414 measured reflections	*l* = −21→23

### Refinement

**Table:**

Refinement on *F*^2^	Primary atom site location: structure-invariant direct methods
Least-squares matrix: full	Secondary atom site location: difference Fourier map
*R*[*F*^2^ > 2σ(*F*^2^)] = 0.025	Hydrogen site location: inferred from neighbouring sites
*wR*(*F*^2^) = 0.060	H atoms treated by a mixture of independent and constrained refinement
*S* = 1.05	*w* = 1/[σ^2^(*F*_o_^2^) + (0.0216*P*)^2^ + 1.9328*P*] where *P* = (*F*_o_^2^ + 2*F*_c_^2^)/3
3007 reflections	(Δ/σ)_max_ = 0.001
165 parameters	Δρ_max_ = 0.36 e Å^−^^3^
1 restraint	Δρ_min_ = −0.51 e Å^−^^3^

### Special details

**Table:**

Geometry. All e.s.d.'s (except the e.s.d. in the dihedral angle between two l.s. planes) are estimated using the full covariance matrix. The cell e.s.d.'s are taken into account individually in the estimation of e.s.d.'s in distances, angles and torsion angles; correlations between e.s.d.'s in cell parameters are only used when they are defined by crystal symmetry. An approximate (isotropic) treatment of cell e.s.d.'s is used for estimating e.s.d.'s involving l.s. planes.
Refinement. Refinement of *F*^2^ against ALL reflections. The weighted *R*-factor *wR* and goodness of fit *S* are based on *F*^2^, conventional *R*-factors *R* are based on *F*, with *F* set to zero for negative *F*^2^. The threshold expression of *F*^2^ > σ(*F*^2^) is used only for calculating *R*-factors(gt) *etc*. and is not relevant to the choice of reflections for refinement. *R*-factors based on *F*^2^ are statistically about twice as large as those based on *F*, and *R*- factors based on ALL data will be even larger.

### Fractional atomic coordinates and isotropic or equivalent isotropic displacement parameters (Å^2^)

**Table:**

	*x*	*y*	*z*	*U*_iso_*/*U*_eq_	
Cu1	0.073508 (14)	0.69663 (3)	0.253261 (16)	0.01063 (8)	
Br1	0.150450 (12)	0.94932 (3)	0.260812 (15)	0.01679 (8)	
O1	0.03282 (8)	0.75649 (19)	0.32448 (10)	0.0119 (3)	
N1	0.14514 (10)	0.5556 (2)	0.34424 (12)	0.0137 (4)	
N2	0.08944 (10)	0.5480 (2)	0.17527 (12)	0.0152 (4)	
C1	0.07547 (12)	0.7880 (3)	0.40514 (14)	0.0117 (4)	
C2	0.05469 (13)	0.9126 (3)	0.44099 (15)	0.0168 (5)	
H2	0.0119	0.9756	0.4073	0.020*	
C3	0.09599 (13)	0.9446 (3)	0.52491 (16)	0.0196 (5)	
H3	0.0810	1.0287	0.5484	0.024*	
C4	0.15940 (13)	0.8552 (3)	0.57543 (15)	0.0190 (5)	
H4	0.1875	0.8778	0.6331	0.023*	
C5	0.18105 (12)	0.7334 (3)	0.54092 (15)	0.0167 (5)	
H5	0.2246	0.6732	0.5755	0.020*	
C6	0.14031 (12)	0.6961 (3)	0.45577 (14)	0.0124 (5)	
C7	0.16441 (12)	0.5626 (3)	0.42169 (15)	0.0145 (5)	
C8	0.21248 (14)	0.4320 (3)	0.48106 (17)	0.0226 (6)	
H8A	0.1951	0.3254	0.4554	0.034*	
H8B	0.2096	0.4381	0.5322	0.034*	
H8C	0.2639	0.4476	0.4941	0.034*	
C9	0.17044 (13)	0.4215 (3)	0.31143 (16)	0.0188 (5)	
H9A	0.1396	0.3240	0.3026	0.023*	
H9B	0.2225	0.3938	0.3514	0.023*	
C10	0.16385 (13)	0.4758 (3)	0.23030 (16)	0.0185 (5)	
H10A	0.2027	0.5571	0.2412	0.022*	
H10B	0.1716	0.3820	0.2021	0.022*	
C11	0.03162 (13)	0.4203 (3)	0.14254 (16)	0.0194 (5)	
H11A	−0.0164	0.4691	0.1044	0.029*	
H11B	0.0290	0.3688	0.1887	0.029*	
H11C	0.0441	0.3386	0.1130	0.029*	
C12	0.09097 (15)	0.6276 (3)	0.10441 (16)	0.0221 (6)	
H12A	0.1035	0.5475	0.0742	0.033*	
H12B	0.1282	0.7140	0.1251	0.033*	
H12C	0.0422	0.6741	0.0670	0.033*	
O2	0.0000	1.1437 (3)	0.2500	0.0347 (7)	
H2O	0.0349 (14)	1.085 (3)	0.257 (2)	0.042*	

### Atomic displacement parameters (Å^2^)

**Table:**

	*U*^11^	*U*^22^	*U*^33^	*U*^12^	*U*^13^	*U*^23^
Cu1	0.00875 (14)	0.01254 (15)	0.01042 (16)	0.00081 (10)	0.00458 (12)	−0.00042 (11)
Br1	0.01330 (12)	0.01715 (13)	0.01898 (14)	−0.00367 (9)	0.00723 (10)	0.00097 (9)
O1	0.0089 (7)	0.0168 (8)	0.0090 (8)	0.0009 (6)	0.0035 (6)	−0.0008 (6)
N1	0.0113 (9)	0.0135 (10)	0.0164 (11)	0.0011 (8)	0.0069 (8)	0.0003 (8)
N2	0.0124 (9)	0.0188 (11)	0.0146 (11)	0.0004 (8)	0.0068 (8)	−0.0015 (8)
C1	0.0090 (10)	0.0151 (12)	0.0110 (11)	−0.0012 (9)	0.0050 (9)	−0.0005 (9)
C2	0.0143 (11)	0.0203 (12)	0.0152 (13)	0.0019 (9)	0.0068 (10)	−0.0009 (10)
C3	0.0194 (12)	0.0242 (14)	0.0182 (13)	−0.0022 (10)	0.0114 (11)	−0.0063 (11)
C4	0.0176 (12)	0.0292 (14)	0.0099 (12)	−0.0067 (10)	0.0066 (10)	−0.0032 (10)
C5	0.0109 (11)	0.0228 (13)	0.0144 (13)	−0.0012 (9)	0.0047 (10)	0.0042 (10)
C6	0.0114 (10)	0.0149 (11)	0.0122 (12)	−0.0020 (9)	0.0068 (9)	0.0014 (9)
C7	0.0088 (10)	0.0151 (12)	0.0184 (13)	0.0003 (9)	0.0057 (10)	0.0037 (10)
C8	0.0238 (13)	0.0204 (14)	0.0224 (14)	0.0074 (11)	0.0105 (12)	0.0074 (11)
C9	0.0161 (12)	0.0170 (12)	0.0203 (13)	0.0050 (10)	0.0067 (11)	−0.0038 (10)
C10	0.0122 (11)	0.0230 (13)	0.0192 (14)	0.0031 (10)	0.0068 (10)	−0.0043 (11)
C11	0.0172 (12)	0.0189 (13)	0.0200 (13)	−0.0019 (10)	0.0075 (11)	−0.0063 (10)
C12	0.0251 (13)	0.0281 (14)	0.0180 (14)	0.0015 (11)	0.0144 (11)	−0.0019 (11)
O2	0.0404 (18)	0.0183 (15)	0.059 (2)	0.000	0.0349 (17)	0.000

### Geometric parameters (Å, °)

**Table:**

Cu1—O1	1.9480 (15)	C5—C6	1.408 (3)
Cu1—N1	1.983 (2)	C5—H5	0.9500
Cu1—O1^i^	2.0138 (15)	C6—C7	1.474 (3)
Cu1—N2	2.042 (2)	C7—C8	1.512 (3)
Cu1—Br1	2.5874 (5)	C8—H8A	0.9800
O1—C1	1.334 (3)	C8—H8B	0.9800
O1—Cu1^i^	2.0138 (15)	C8—H8C	0.9800
N1—C7	1.287 (3)	C9—C10	1.508 (4)
N1—C9	1.478 (3)	C9—H9A	0.9900
N2—C12	1.482 (3)	C9—H9B	0.9900
N2—C11	1.483 (3)	C10—H10A	0.9900
N2—C10	1.492 (3)	C10—H10B	0.9900
C1—C2	1.402 (3)	C11—H11A	0.9800
C1—C6	1.422 (3)	C11—H11B	0.9800
C2—C3	1.381 (3)	C11—H11C	0.9800
C2—H2	0.9500	C12—H12A	0.9800
C3—C4	1.392 (4)	C12—H12B	0.9800
C3—H3	0.9500	C12—H12C	0.9800
C4—C5	1.379 (3)	O2—H2O	0.827 (17)
C4—H4	0.9500		
			
O1—Cu1—N1	88.11 (7)	C5—C6—C1	118.2 (2)
O1—Cu1—O1^i^	74.57 (7)	C5—C6—C7	120.0 (2)
N1—Cu1—O1^i^	148.18 (7)	C1—C6—C7	121.8 (2)
O1—Cu1—N2	155.03 (7)	N1—C7—C6	121.8 (2)
N1—Cu1—N2	86.20 (8)	N1—C7—C8	120.7 (2)
O1^i^—Cu1—N2	98.28 (7)	C6—C7—C8	117.6 (2)
O1—Cu1—Br1	102.77 (5)	C7—C8—H8A	109.5
N1—Cu1—Br1	104.03 (6)	C7—C8—H8B	109.5
O1^i^—Cu1—Br1	105.71 (5)	H8A—C8—H8B	109.5
N2—Cu1—Br1	102.19 (6)	C7—C8—H8C	109.5
C1—O1—Cu1	122.55 (13)	H8A—C8—H8C	109.5
C1—O1—Cu1^i^	137.48 (13)	H8B—C8—H8C	109.5
Cu1—O1—Cu1^i^	98.14 (7)	N1—C9—C10	108.1 (2)
C7—N1—C9	120.9 (2)	N1—C9—H9A	110.1
C7—N1—Cu1	127.71 (16)	C10—C9—H9A	110.1
C9—N1—Cu1	111.06 (15)	N1—C9—H9B	110.1
C12—N2—C11	108.61 (19)	C10—C9—H9B	110.1
C12—N2—C10	108.36 (18)	H9A—C9—H9B	108.4
C11—N2—C10	110.64 (19)	N2—C10—C9	110.74 (18)
C12—N2—Cu1	116.21 (15)	N2—C10—H10A	109.5
C11—N2—Cu1	109.58 (14)	C9—C10—H10A	109.5
C10—N2—Cu1	103.31 (14)	N2—C10—H10B	109.5
O1—C1—C2	118.9 (2)	C9—C10—H10B	109.5
O1—C1—C6	121.6 (2)	H10A—C10—H10B	108.1
C2—C1—C6	119.4 (2)	N2—C11—H11A	109.5
C3—C2—C1	120.5 (2)	N2—C11—H11B	109.5
C3—C2—H2	119.7	H11A—C11—H11B	109.5
C1—C2—H2	119.7	N2—C11—H11C	109.5
C2—C3—C4	120.8 (2)	H11A—C11—H11C	109.5
C2—C3—H3	119.6	H11B—C11—H11C	109.5
C4—C3—H3	119.6	N2—C12—H12A	109.5
C5—C4—C3	119.4 (2)	N2—C12—H12B	109.5
C5—C4—H4	120.3	H12A—C12—H12B	109.5
C3—C4—H4	120.3	N2—C12—H12C	109.5
C4—C5—C6	121.7 (2)	H12A—C12—H12C	109.5
C4—C5—H5	119.1	H12B—C12—H12C	109.5
C6—C5—H5	119.1		

Symmetry codes: (i) −*x*, *y*, −*z*+1/2.

### Hydrogen-bond geometry (Å, °)

**Table:**

*D*—H···*A*	*D*—H	H···*A*	*D*···*A*	*D*—H···*A*
C11—H11B···O2^ii^	0.98	2.40	3.299 (3)	152
O2—H2O···Br1	0.83 (2)	2.62 (2)	3.4269 (14)	167 (3)

Symmetry codes: (ii) *x*, *y*−1, *z*.
